# The diversity of opinion among general practitioners regarding the threat and measures against COVID-19 – Cross-sectional survey

**DOI:** 10.1080/13814788.2021.1954155

**Published:** 2021-07-28

**Authors:** Klaus Linde, Christian Bergmaier, Marion Torge, Niklas Barth, Antonius Schneider, Alexander Hapfelmeier

**Affiliations:** Institute of General Practice and Health Services Research, Technical University Munich, Munich, Germany

**Keywords:** COVID-19, infectious diseases, attitudes, general practice, survey

## Abstract

**Background:**

After the ‘first wave’ in spring 2020, opinions regarding the threat and measures against COVID-19 seemed to vary among German general practitioners (GPs).

**Objectives:**

To systematically investigate opinions and to identify subgroups of GPs sharing similar views.

**Methods:**

A questionnaire was sent to all 210 practices accredited for undergraduate teaching of family medicine at the Medical Faculty of the Technical University of Munich. Questions addressed personal opinions regarding risks, dilemmas, restrictions and their relaxation associated with COVID-19, and personal fears, symptoms of depression and anxiety. Patterns of strong opinions (‘archetypes’) were identified using archetypal analysis, a statistical method seeking extremal points in the multidimensional data.

**Results:**

One hundred and sixty-one GPs sent back a questionnaire (response rate 77%); 143 (68%) with complete data for all 38 relevant variables could be included in the analysis. We identified four archetypes with subgroups of GPs tending in the direction of these archetypes: a small group of ‘Sceptics’ (*n* = 12/8%) considering threats of COVID-19 as overrated and measures taken as exaggerated; ‘Hardliners’ (*n* = 34/24%) considering threats high and supporting strong measures; ‘Balancers’ (*n* = 77/54%) who also rated the threats high but were more critical about potentially impairing the quality of life of elderly people and children; and ‘Anxious’ GPs (*n* = 20/14%) tending to report more fear, depressive and anxiety symptoms.

**Conclusion:**

Among the participants in this survey, opinions regarding the threat and the measures taken against COVID-19 during the ‘first wave’ in Germany in spring 2020 varied greatly.

KEY MESSAGES

- After the ‘first wave’ in spring 2020, opinions regarding the threat and measures against COVID-19 varied greatly among German general practitioners.
- Four types of opinion patterns could be identified: Corona ‘Sceptics’, ‘Hardliners’, ‘Balancers’, and ‘Anxious’.
- Actively studying the opinions of frontline doctors helps to understand controversies in the profession.

## Introduction

The ‘first wave’ of the COVID-19 pandemic in the spring of 2020 posed major challenges to general practice in many countries regarding safeguards, workload, organisation, and on a financial level [1–3]. At that time, it seemed that a large majority in the medical profession agreed that COVID-19 is a relevant threat and that strong measures are justifiable to minimise the death toll and to limit other harmful consequences. However, there was a considerable debate early on about the scale of the threat and the most appropriate measures to take [4]. It seems likely that different opinions also existed among GPs regarding the threat and the measures taken against COVID-19.

Compared to other large Western industrial countries, Germany was hit relatively mildly by the first wave of the pandemic. By 30 June 2020, the cumulative number of verified COVID-19 cases in Germany (total population 83 million) was 194,259; the number of related deaths was 8,973 [5]. For example, in the UK (67 million), these figures were 284,097 and 40,639, respectively [6]. But despite a relatively liberal ‘lockdown’, a small minority of the German population actively protested against the measures taken by the government, sometimes negating that COVID-19 is a relevant threat at all [7,8]. In this movement, conspiracy theories and far-right populist views had an important role but also mixed with left-wing system criticism, vaccine-scepticism, et cetera [9,10]. While the number of medical doctors publicly supporting this movement was very small, their ‘expert status’ served to give ‘authority’ to deviant views [11]. Alongside these apparent radical positions, there was much more reasonable but sometimes vigorous debate among GPs about the adequate way to cope with the pandemic, for example, in the restricted email list of the German College of General Practitioners and Family Physicians (‘Listserver Allgemeinmedizin’).

A hypothetical scenario of a pandemic with a SARS virus, which was developed for the German government in 2012, predicted that such an event would be assessed inconsistently by experts, increasing uncertainty among the population [12]. With their central role as primary care providers, GPs can have an essential influence on the views and behaviour of their patients [13]. Knowing opinions of frontline GPs about the pandemic and its management is vital to estimate the risk of misinformation and for developing strategies to minimise such risk in future pandemics. We performed a cross-sectional survey among Bavarian GPs to investigate the opinions regarding the threat and the measures taken against COVID-19. In particular, we aimed to identify the variety and range of opinions and subgroups of GPs sharing similar views.

## Methods

### Design and sample

The study was an anonymous cross-sectional survey approved by the ethics committee of the Medical Faculty of the Technical University of Munich (file number 334/20S). On 17 June 2020, a four-page questionnaire was sent by mail to all 210 contact GPs of practices participating in the undergraduate teaching network for family medicine of the Institute of General Practice and Health Services Research. Reminders were sent three and six weeks later. To be accredited as teaching practices, general practitioners’ practices must provide typical, unselected primary care in the community for those making initial contact with a health care professional within the social health insurance system (covering about 90% of the German population) and host regularly (about once per academic half-year) medical students for the obligatory 2-weeks family medicine trainee-ship. A third of the practices are located in the city of Munich or within reach of the local public transport association (approximately 20 miles around the city). The remaining practices are located in villages and towns, mostly in southern Bavaria (6.8 million inhabitants).

### Questionnaire

The authors developed the questionnaire with further support from four practising, independent GPs. It consisted of 52 items with eleven blocks. Four blocks covered characteristics of respondents and their practices; the number of COVID-19 cases, hospitalisations and deaths in practice and nursing homes; infections of practice personnel; and challenges for the practice. A descriptive analysis of the findings from this part has been published in German [14]. The six blocks being the main focus in this paper comprised statements regarding personal views on the threats posed by COVID-19 (4 statements); the measures taken by the German government in March 2020 (7 statements); the relaxation of measures in May 2020 (5 statements); the basic dilemma of the ‘costs’ of saving lives (4 statements); consequences for the coming months (6 statements); and concerns regarding infections in the practice and economic consequences (5 statements). Agreement was rated on a five-point Likert scale ranging from −2 = do not agree to 2 = agree. Participants could add comments in free-text fields after each of the six blocks. The final block consisted of the PHQ-4, a validated, ultra-brief screening scale for anxiety and depression [15].

### Analysis

Descriptive statistics were computed to present the distribution of the data. We used a statistical method called ‘archetypal analysis’ to describe the range of opinions [16]. An archetype is defined as ‘something that is considered to be a perfect or typical example of a particular kind of person or thing because it has all their most important characteristics’ [17]. Technically, the archetype is represented by extremal points on the convex hull of the observed multivariable data, which is given by the answers to the seven blocks of questions that have been outlined above. The optimal number of archetypes was chosen by the ‘elbow criterion’, that is by the number of archetypes beyond which the sum of squared differences (i.e. the residual sum of squares) between the given observations and the archetypes did not decisively improve any more [18]. Single observations were then allocated to the closest archetype to define respective subgroups. The archetypes were named based on their most characteristic properties. Analysis was performed using IBM SPSS Statistics 25 (Armonk, NY) and R 4.0.3 (The R Foundation for Statistical Computing, Vienna, Austria). The ‘archetypal analysis’ was conducted by use of the R-package ‘archetypes’ [18].

## Results

Until 31 August 2020, a total of 161 GPs (77%) answered the questionnaire. As 18 (9%) had one or more missing values in the 38 relevant variables, 143 (68%) could be included in the statistical analysis. Among these, 52 (35%) were female, 66 (46%) were 51–60 years, and 34 (18%) were older than 60 years. Fifty-eight (42%) reported that their district was an infection ‘hotspot’. GPs had cared for a median (25th and 75th percentile) of 10 (4 and 20) COVID-19 patients (Table 1). Twelve (8%) participants had not yet seen a COVID-19 patient in their practice.

**Table 1.** Characteristics of participants/practices and experiences with COVID-19 in practices and nursing homes. Figures presented are absolute frequencies (percentages) or medians (25th/75th percentiles).

Variable (number of missing values among all participants/subgroups)	All *N* = 143	Sceptics *n* = 12	Hardliners *n* = 34	Balancers *n* = 77	Anxious *n* = 20
Characteristics of participants/practices					
Female (0)	52 (35%)	3 (25%)	12 (35%)	32 (42%)	5 (25%)
Ag*e* > 60 years (0)	34 (24%)	6 (50%)	10 (29%)	15 (20%)	3 (15%)
In own practice since more than 20 years (2/0/1/1/0)	55 (39%)	8 (67%)	14 (42%)	27 (36%)	6 (30%)
Rural practice location (0)	69 (48%)	7 (58%)	15 (44%)	39 (51%)	8 (40%)
Practice close to a Covid-19 hotspot (4/0/1/3/0)	58 (42%)	4 (33%)	17 (52%)	27 (37%)	10 (50%)
At least one member of the practice team had SARS-CoV-2 infection (0)	29 (20%)	2 (17%)	8 (24%)	13 (17%)	6 (30%)
Had to introduce short-time work in the practice (0)	27 (19%)	2 (17%)	3 (9%)	15 (20%)	7 (35%)
COVID-19 in the practice					
Number of patients with SARS-CoV-2 infection in practice (8/0/2/2/5)	10 (4/20)	4 (0/18)	10 (6/18)	10 (4/20)	14 (3/20)
Number of COVID-19 tests performed in practice (45/2/13/23/7)*	53 (20/100)	10 (4/43)	35 (20/95)	74 (29/102)	40 (30/156)
Number of patients hospitalised due to COVID-19 (5/0/0/2/3)*	1 (0/2)	0 (0/2)	1 (0/3)	1 (0/2)	1 (2/4)
Number of patients died from COVOD-19 (3/0/0/2/1)	0 (0/1)	0 (0/1)	0 (0/1)	0 (0/1)	0 (0/1)
COVID-19 in nursing homes					
GPs caring for a nursing home (0)	121 (85%)	12 (100%)	29 (85%)	64 (83%)	16 (80%)
Nursing homes at least SARS-CoV-2 infection (0)§	27 (22%)	2 (17%)	12 (41%)	7 (11%)	6 (37%)
At least 5 patients hospitalised (0)$	9	1	4	1	3
At least 3 patients died (0)$	6	0	3	1	2

*p-value in comparison between subgroups (from Kruskal-Wallis-test or χ^2^ test) < 0.05; § analysis without the 22 GPs not caring for a nursing home; $only for the 27 nursing homes with COVID-19 cases.

The whole range of answer options from strong agreement to strong disagreement was chosen for 28 of the 30 statements regarding personal concerns, the threats posed by COVID-19, the basic dilemma, the measures taken in March 2020, the relaxation phase in May 2020, and the consequences for the coming months. The 10th to the 90th percentile ranged across all five answer options for 7 items, across four answer options for 14 items and across three answer options for five items, reflecting the diversity of answers to specific questions. Strong agreement was observed only for the request to keep your distance (94% strongly agree + 4% agree), the ban on major events (97%+2%) and the exit restrictions (81%+12%) in March, and the request to keep your distance in May (79%+12%).

**Figure 1.**
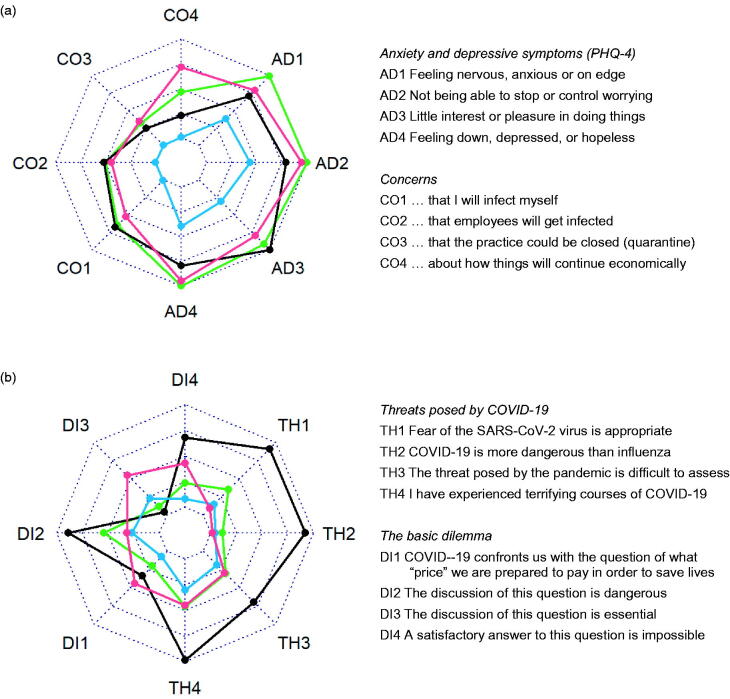
Radar charts showing the answer patterns of the four **archetpyes** (black = **sceptics**, red = **hardliners**, green = **balancers**, and blue = **anxious**) regarding anxiety and depressive symptoms and concerns (a), and for threat posed by COVID-19 and the basic dilemma (b). The innermost circles indicate strong approval, the outermost circles strong disagreement.

**Figure 2.**
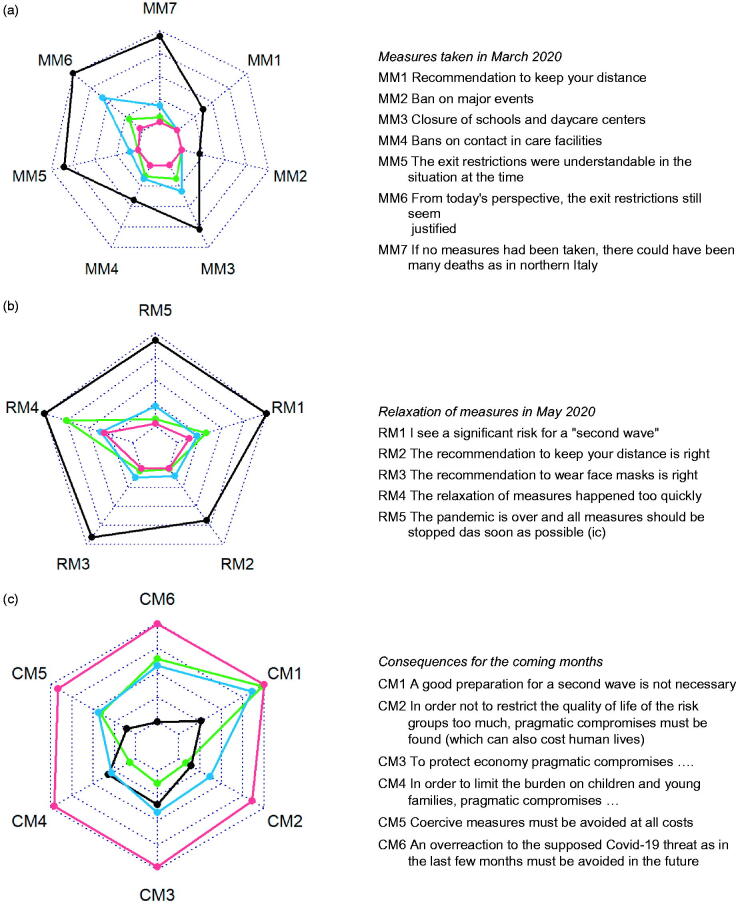
Radar charts showing the answer patterns of the four **archetpyes** (black =**sceptics**, red = **hardliners**, green = **balancers**, and blue = **anxious**) regarding measures taken in March 2020 (a), relaxations in May 2020 (b), and consequences for the coming months (c). The innermost circles indicate strong approval, the outermost circles strong disagreement. For reasons of readability some items were inversely coded (= ic) or reworded.

Four archetypes were identified: the ‘Sceptic’, the ‘Hardliner’, the ‘Balancer’, and the ‘Anxious’ (see Supplementary eFigure1 for information on model fit). Their response patterns are displayed in Figures 1 and 2. When referring to the individual archetypes we will use the pronoun ‘they’ in its singular, gender-neutral form. The ’Sceptic‘ held the most distinct opinion: In early summer 2020, they considered the fear of COVID-19 as inappropriate and the threat quite comparable to that of influenza (Figure 1(b)). Their agreement to the measures taken in March 2020 was less than the other three archetypes. While they considered the ban of major events and the recommendation to keep distance as at least somewhat justified, they were ambiguous regarding the closure of schools and the contact ban in care facilities and critical about the exit restrictions (Figure 2(a)). They saw little risk for a ‘second wave’, tended to think that the pandemic is over and that all measures should be stopped (Figure 2(b)). In their view, an overreaction to the supposed threat by COVID-19 as in spring 2020 had to be avoided in winter 2020/21.

The second archetype emerging from the analysis was the ’Hardliner’. Their most striking characteristics were that they strongly agreed with all measures taken in spring (Figure 2(a and b)). They pleaded for taking strict measures in the winter of 2020/21 to save lives, even if this should lead to other relevant social harm (Figure 2(c)).

The third archetype, the ‘Balancer’, was similar to the ’Hardliner‘ and the ’Anxious‘ in several aspects. However, they seemed particularly keen to balance the goal of saving lives with other societal harms. Therefore, for winter 2020/21, they were clearly favouring pragmatic compromises to avoid restricting the quality of life in risks groups too much, putting too much burden on children and young families, or harming the economy too severely (Figure 2(c)).

Finally, the ‘Anxious‘, mainly was characterised by somewhat higher levels for anxiety and depressive symptoms on the PHQ-4 and the fear of COVID-19 infection in their practice (Figure 1(a)). Regarding threats and measures they were similar to balancers.

All GPs ranged within the aforementioned strong opinions of the archetypes. Attributing each GP to the closest archetype leads to the classification of twelve GPs (8%) as ’Sceptics’, 34 (24%) as ’Hardliners’, 77 (54%) as ‘Balancers’, and 20 (14%) as ’Anxious’. ’Sceptics’ tended to be more often highly experienced, to have done less PCR-testing for COVID-19, and to have seen severe cases less frequently (Table 1). The average response patterns of the subgroups are displayed in Supplementary eFigures 2 and 3, and eTable 1.

## Discussion

### Main findings

Among the participants in this survey, opinions regarding the threat and the measures taken against COVID-19 during the ‘first wave’ in spring 2020 varied greatly. Analysis identified four archetypes with subgroups of GPs leaning in the direction of respective strong opinions: a small group of ’Sceptics’ considering threats of COVID-19 as overrated and measures taken exaggerated; ’Hardliners’ considering threats high and supporting strong measures; ‘Balancers’ who also tended to consider threats high but were more critical about measures potentially impairing the quality of life of elderly people and children, and ’Anxious’ GPs tending to report more fears, depressive and anxiety symptoms.

### Strengths and limitations

While several studies have investigated the psychological impact of COVID-19 on health care workers, including one study among Italian GPs [19,20], we have not been able to identify any research on the main subject of our study among physicians at the time of writing (June 2021). The sample size of our study is small. Therefore, the lack of statistically significant associations between archetypal subgroups and variables such as age, gender, practice location or number of COVID-19 patients seen does not mean that such associations do not exist. The response rate in our survey is high but we sent the questionnaire to a convenience sample rather than a random sample. While our participants run typical front line primary care practices, they are probably not representative for Bavarian or German GPs in every respect. Archetypal analysis is an exploratory, data-driven statistical method which, in our case, provides models of strong opinions in a specific group of GPs in a specific region at a specific point in time. At the time our study was carried out, Germany was relatively little affected by the pandemic. If our study had been done in the badly affected region of Piedmont, Italy, where many doctors died [21], we would not have dared to ask some of our questions. Furthermore, to keep our questionnaire short, we could not ask for further aspects probably influencing the participants’ views (e.g. missed care among patients with chronic diseases or mental problems, health inequalities etc.). These issues were addressed in an additional qualitative study, which is still under analysis. Despite these limitations, we think that our archetypes provide a crude but intuitive and straightforward classification of basic opinion patterns among German GPs. While the size of subgroups could well have been different in a random sample, it seems unlikely that random sampling would have altered the definition of archetypes per se. The archetypes might have been valid to some extent also for the second and third wave of the pandemic in Germany (November 2020 to January 2021 and March to May 2021). Reports on ‘Corona-skeptic’ GPs still appeared in the media, and discussion among GPs with ‘Hardliner’ and ‘Balancer’ positions clearly went on. An ‘Anxious’ subgroup has been less visible in the public discussion. Still, the multiple studies on the psychological impact of the pandemic and the measures taken suggest that such a subgroup might still exist (e.g. [22]). Our archetypes might also apply to doctors in other countries, but the frequency of subgroups is likely to differ.

### Interpretation

We have to emphasise again that our archetypes are extreme ‘models’ and that most real GPs hold opinions that are less extreme, often variable depending on the specific issue and sometimes contradictory. For example, there was only one ‘skeptical’ GP in our survey who was extreme on most issues and (as his free-text comments suggest) seemed to agree with conspiracy views. Most ‘sceptics’ agreed to the ban on major events and the call to keep your distance but (in early summer 2020) took the view that the pandemic is over and the threat had been overestimated. It is unclear what these GPs think today. While their opinion might have been over-optimistic and highly questionable according to the scientific knowledge already available at that time, they did not seem to hold conspiracist views.

Freedom of opinion is a fundamental good in democratic societies, and open controversy is an important principle in science. The diverse views among ‘hardliners’, ‘balancers’, ‘anxious’ and even among slightly ‘skeptical’ GPs must be legitimate and tolerated. However, extreme and conspiracist views among medical doctors are a matter of concern in the pandemic. Tagliabue et al., lament a ‘pandemic of misinformation’ that is causing confusion and uncertainty among the population [23]. Articles in major medical journals see the role of medical doctors in tackling fake news and providing reliable information [24,25]. However, there is very little information to what extent health care workers in general and GPs in particular share or disagree with ‘mainstream’ opinions, and to what extent they contribute to misinformation. For example, vaccine hesitancy among physicians is a considerable problem [26], which can impact on the acceptance of COVID-19 vaccines in the general population. An article in JAMA discusses the role of prominent physicians and researchers in actively disseminating harmful recommendations and affirms that, while academic freedom must be respected, physicians and scientists have a professional obligation to respond when science is being ‘mis-represented’ [27].

A part of the diversity of opinions found in our study might also be due to the lack of involvement of GPs (and other relevant stakeholders beyond virologists and epidemiologists) into committees advising German politicians on anti-pandemic measures and suboptimal information management. In a public statement in December 2020, the German College of General Practitioners and Family Physicians (DEGAM) complained that to act as a multiplier, GPs must know the rationale based on which political measures were taken [28]. Given the central role of primary care it seems important to involve and to inform GPs better to ensure good implementation of relevant decisions and measures.

## Conclusion

Among the participants in this survey, opinions regarding the threat and measures taken against COVID-19 during the ‘first wave’ in Germany in spring 2020 varied greatly. Adequate communication of the rationale of political decisions and, if possible, involvement of GPs in decision-making processes relevant to primary care might reduce major dissent and facilitate implementation of measures in the future.

## Supplementary Material

Supplemental Material: eTable 1

Supplemental Material: eFigures 2-3

Supplemental Material: eFigure 1
