# Supplementary material for: The diversity of opinion among general practitioners regarding the threat and measures against COVID-19 – Cross-sectional survey
Source: Eur J Gen Pract. 2021 Jul 28;27(1):176–83. doi: 10.1080/13814788.2021.1954155 (PMC8330783; doi:10.1080/13814788.2021.1954155)
Supplement: Supplemental Material: eTable 1 [file IGEN_A_1954155_SM2202.docx]

Appendix eTable 1

Summary of responses to the single items. P-values from analysis of variance. For explanation of the item abbreviations see Figures 1 and 2 or eFigures 2 and 3)

|  | **Balancers** | **Skeptics** | **Anxious** | **Hardliners** | **All** |  |
| --- | --- | --- | --- | --- | --- | --- |
|  | **(N=77)** | **(N=12)** | **(N=20)** | **(N=34)** | **N=143)** | **p value** |
| **AD1** |  |  |  |  |  | < 0.001 |
| Mean (SD) | 3.71 (0.48) | 3.42 (0.90) | 2.55 (0.89) | 3.47 (0.51) | 3.47 (0.71) |  |
| Range | 2.00 - 4.00 | 1.00 - 4.00 | 1.00 - 4.00 | 3.00 - 4.00 | 1.00 - 4.00 |  |
| **AD2** |  |  |  |  |  | < 0.001 |
| Mean (SD) | 3.86 (0.35) | 3.58 (0.90) | 2.70 (0.66) | 3.74 (0.45) | 3.64 (0.62) |  |
| Range | 3.00 - 4.00 | 1.00 - 4.00 | 1.00 - 4.00 | 3.00 - 4.00 | 1.00 - 4.00 |  |
| **AD3** |  |  |  |  |  | < 0.001 |
| Mean (SD) | 3.51 (0.55) | 3.58 (0.67) | 2.65 (0.75) | 3.24 (0.55) | 3.33 (0.66) |  |
| Range | 2.00 - 4.00 | 2.00 - 4.00 | 1.00 - 4.00 | 2.00 - 4.00 | 1.00 - 4.00 |  |
| **AD4** |  |  |  |  |  | < 0.001 |
| Mean (SD) | 3.94 (0.25) | 3.67 (0.89) | 2.70 (0.98) | 3.79 (0.41) | 3.71 (0.66) |  |
| Range | 3.00 - 4.00 | 1.00 - 4.00 | 1.00 - 4.00 | 3.00 - 4.00 | 1.00 - 4.00 |  |
| **CO1** |  |  |  |  |  | < 0.001 |
| Mean (SD) | 3.09 (1.32) | 3.08 (1.51) | 1.45 (0.69) | 2.82 (1.42) | 2.80 (1.40) |  |
| Range | 1.00 - 5.00 | 1.00 - 5.00 | 1.00 - 3.00 | 1.00 - 5.00 | 1.00 - 5.00 |  |
| **CO2** |  |  |  |  |  | 0.003 |
| Mean (SD) | 2.38 (1.17) | 2.75 (1.36) | 1.40 (0.60) | 2.38 (1.23) | 2.27 (1.19) |  |
| Range | 1.00 - 5.00 | 1.00 - 5.00 | 1.00 - 3.00 | 1.00 - 5.00 | 1.00 - 5.00 |  |
| **CO3** |  |  |  |  |  | 0.016 |
| Mean (SD) | 2.03 (1.16) | 1.92 (1.16) | 1.30 (0.80) | 2.35 (1.30) | 1.99 (1.18) |  |
| Range | 1.00 - 4.00 | 1.00 - 4.00 | 1.00 - 4.00 | 1.00 - 5.00 | 1.00 - 5.00 |  |
| **CO4** |  |  |  |  |  | 0.002 |
| Mean (SD) | 2.55 (1.35) | 2.25 (1.22) | 1.65 (0.99) | 3.06 (1.32) | 2.52 (1.35) |  |
| Range | 1.00 - 5.00 | 1.00 - 4.00 | 1.00 - 4.00 | 1.00 - 5.00 | 1.00 - 5.00 |  |
| **TH1** |  |  |  |  |  | < 0.001 |
| Mean (SD) | 2.42 (1.14) | 3.92 (0.79) | 1.80 (0.77) | 1.91 (1.26) | 2.34 (1.22) |  |
| Range | 1.00 - 5.00 | 3.00 - 5.00 | 1.00 - 3.00 | 1.00 - 5.00 | 1.00 - 5.00 |  |
| **TH2** |  |  |  |  |  | < 0.001 |
| Mean (SD) | 1.78 (1.19) | 3.42 (1.31) | 1.75 (1.16) | 1.59 (0.92) | 1.87 (1.22) |  |
| Range | 1.00 - 5.00 | 2.00 - 5.00 | 1.00 - 5.00 | 1.00 - 5.00 | 1.00 - 5.00 |  |
| **TH3** |  |  |  |  |  | 0.275 |
| Mean (SD) | 2.31 (1.17) | 2.92 (1.08) | 2.05 (1.19) | 2.26 (1.38) | 2.31 (1.22) |  |
| Range | 1.00 - 5.00 | 1.00 - 5.00 | 1.00 - 5.00 | 1.00 - 5.00 | 1.00 - 5.00 |  |
| **TH4** |  |  |  |  |  | 0.001 |
| Mean (SD) | 2.92 (1.68) | 4.25 (1.22) | 2.30 (1.59) | 2.29 (1.34) | 2.80 (1.63) |  |
| Range | 1.00 - 5.00 | 1.00 - 5.00 | 1.00 - 5.00 | 1.00 - 5.00 | 1.00 - 5.00 |  |
| **DI1** |  |  |  |  |  | 0.035 |
| Mean (SD) | 1.81 (0.99) | 2.58 (1.31) | 1.70 (0.92) | 2.29 (1.49) | 1.97 (1.17) |  |
| Range | 1.00 - 5.00 | 1.00 - 5.00 | 1.00 - 4.00 | 1.00 - 5.00 | 1.00 - 5.00 |  |
| **DI2** |  |  |  |  |  | 0.003 |
| Mean (SD) | 3.22 (1.52) | 3.58 (1.08) | 2.20 (1.24) | 2.41 (1.54) | 2.92 (1.52) |  |
| Range | 1.00 - 5.00 | 2.00 - 5.00 | 1.00 - 5.00 | 1.00 - 5.00 | 1.00 - 5.00 |  |
| **DI3** |  |  |  |  |  | < 0.001 |
| Mean (SD) | 1.68 (0.94) | 1.83 (0.94) | 2.00 (0.79) | 2.74 (1.50) | 1.99 (1.16) |  |
| Range | 1.00 - 5.00 | 1.00 - 4.00 | 1.00 - 3.00 | 1.00 - 5.00 | 1.00 - 5.00 |  |
| **DI4** |  |  |  |  |  | 0.017 |
| Mean (SD) | 2.12 (1.28) | 2.92 (1.24) | 1.65 (0.75) | 2.59 (1.58) | 2.23 (1.33) |  |
| Range | 1.00 - 5.00 | 1.00 - 5.00 | 1.00 - 3.00 | 1.00 - 5.00 | 1.00 - 5.00 |  |
| **MM1** |  |  |  |  |  | < 0.001 |
| Mean (SD) | 1.01 (0.11) | 1.92 (1.24) | 1.00 (0.00) | 1.03 (0.17) | 1.09 (0.44) |  |
| Range | 1.00 - 2.00 | 1.00 - 5.00 | 1.00 - 1.00 | 1.00 - 2.00 | 1.00 - 5.00 |  |
| **MM2** |  |  |  |  |  | < 0.001 |
| Mean (SD) | 1.00 (0.00) | 1.58 (1.16) | 1.00 (0.00) | 1.00 (0.00) | 1.05 (0.36) |  |
| Range | 1.00 - 1.00 | 1.00 - 5.00 | 1.00 - 1.00 | 1.00 - 1.00 | 1.00 - 5.00 |  |
| **MM3** |  |  |  |  |  | < 0.001 |
| Mean (SD) | 1.81 (1.03) | 3.50 (1.09) | 2.05 (1.10) | 1.24 (0.55) | 1.85 (1.10) |  |
| Range | 1.00 - 5.00 | 2.00 - 5.00 | 1.00 - 4.00 | 1.00 - 3.00 | 1.00 - 5.00 |  |
| **MM4** |  |  |  |  |  | < 0.001 |
| Mean (SD) | 1.69 (0.86) | 2.50 (1.09) | 1.65 (1.04) | 1.21 (0.54) | 1.64 (0.90) |  |
| Range | 1.00 - 4.00 | 1.00 - 4.00 | 1.00 - 5.00 | 1.00 - 3.00 | 1.00 - 5.00 |  |
| **MM5** |  |  |  |  |  | < 0.001 |
| Mean (SD) | 1.13 (0.38) | 3.25 (1.22) | 1.25 (0.44) | 1.06 (0.24) | 1.31 (0.76) |  |
| Range | 1.00 - 3.00 | 1.00 - 5.00 | 1.00 - 2.00 | 1.00 - 2.00 | 1.00 - 5.00 |  |
| **MM6** |  |  |  |  |  | < 0.001 |
| Mean (SD) | 2.29 (1.33) | 4.58 (0.51) | 3.00 (1.21) | 1.68 (0.98) | 2.43 (1.40) |  |
| Range | 1.00 - 5.00 | 4.00 - 5.00 | 1.00 - 5.00 | 1.00 - 5.00 | 1.00 - 5.00 |  |
| **MM7** |  |  |  |  |  | < 0.001 |
| Mean (SD) | 1.60 (0.96) | 3.67 (1.07) | 1.50 (0.69) | 1.26 (0.57) | 1.68 (1.05) |  |
| Range | 1.00 - 5.00 | 2.00 - 5.00 | 1.00 - 3.00 | 1.00 - 3.00 | 1.00 - 5.00 |  |
| **RM1** |  |  |  |  |  | < 0.001 |
| Mean (SD) | 2.60 (1.05) | 4.33 (0.78) | 1.75 (0.97) | 1.97 (1.00) | 2.48 (1.20) |  |
| Range | 1.00 - 5.00 | 3.00 - 5.00 | 1.00 - 4.00 | 1.00 - 5.00 | 1.00 - 5.00 |  |
| **RM2** |  |  |  |  |  | < 0.001 |
| Mean (SD) | 1.27 (0.62) | 2.75 (1.22) | 1.20 (0.70) | 1.09 (0.29) | 1.34 (0.77) |  |
| Range | 1.00 - 4.00 | 1.00 - 5.00 | 1.00 - 4.00 | 1.00 - 2.00 | 1.00 - 5.00 |  |
| **RM3** |  |  |  |  |  | < 0.001 |
| Mean (SD) | 1.52 (0.94) | 3.25 (1.29) | 1.40 (0.75) | 1.24 (0.50) | 1.58 (1.00) |  |
| Range | 1.00 - 4.00 | 1.00 - 5.00 | 1.00 - 4.00 | 1.00 - 3.00 | 1.00 - 5.00 |  |
| **RM4** |  |  |  |  |  | < 0.001 |
| Mean (SD) | 3.87 (0.92) | 4.58 (0.67) | 2.65 (1.18) | 2.85 (1.10) | 3.52 (1.16) |  |
| Range | 1.00 - 5.00 | 3.00 - 5.00 | 1.00 - 5.00 | 1.00 - 5.00 | 1.00 - 5.00 |  |
| **RM5** |  |  |  |  |  | < 0.001 |
| Mean (SD) | 1.48 (0.87) | 3.75 (1.14) | 1.80 (1.15) | 1.21 (0.48) | 1.65 (1.08) |  |
| Range | 1.00 - 5.00 | 2.00 - 5.00 | 1.00 - 5.00 | 1.00 - 3.00 | 1.00 - 5.00 |  |
| **CM1** |  |  |  |  |  | < 0.001 |
| Mean (SD) | 4.66 (0.62) | 2.92 (1.16) | 4.60 (0.68) | 4.82 (0.58) | 4.55 (0.84) |  |
| Range | 2.00 - 5.00 | 1.00 - 5.00 | 3.00 - 5.00 | 2.00 - 5.00 | 1.00 - 5.00 |  |
| **CM2** |  |  |  |  |  | < 0.001 |
| Mean (SD) | 1.77 (0.74) | 2.25 (0.75) | 2.35 (0.99) | 3.65 (1.12) | 2.34 (1.16) |  |
| Range | 1.00 - 3.00 | 1.00 - 3.00 | 1.00 - 5.00 | 1.00 - 5.00 | 1.00 - 5.00 |  |
| **CM3** |  |  |  |  |  | < 0.001 |
| Mean (SD) | 2.12 (0.90) | 2.75 (1.14) | 2.55 (0.89) | 4.09 (0.75) | 2.70 (1.19) |  |
| Range | 1.00 - 4.00 | 1.00 - 5.00 | 1.00 - 5.00 | 3.00 - 5.00 | 1.00 - 5.00 |  |
| **CM4** |  |  |  |  |  | < 0.001 |
| Mean (SD) | 1.97 (0.92) | 2.50 (1.31) | 2.15 (0.93) | 3.85 (0.96) | 2.49 (1.23) |  |
| Range | 1.00 - 4.00 | 1.00 - 5.00 | 1.00 - 5.00 | 1.00 - 5.00 | 1.00 - 5.00 |  |
| **CM5** |  |  |  |  |  | < 0.001 |
| Mean (SD) | 2.69 (1.36) | 2.08 (1.00) | 3.10 (1.12) | 3.79 (1.27) | 2.96 (1.37) |  |
| Range | 1.00 - 5.00 | 1.00 - 4.00 | 1.00 - 5.00 | 1.00 - 5.00 | 1.00 - 5.00 |  |
| **CM6** |  |  |  |  |  | < 0.001 |
| Mean (SD) | 3.43 (1.39) | 1.92 (1.08) | 3.65 (1.42) | 4.38 (1.04) | 3.56 (1.43) |  |
| Range | 1.00 - 5.00 | 1.00 - 4.00 | 1.00 - 5.00 | 1.00 - 5.00 | 1.00 - 5.00 |  |
