# Supplementary material for: The diversity of opinion among general practitioners regarding the threat and measures against COVID-19 – Cross-sectional survey
Source: Eur J Gen Pract. 2021 Jul 28;27(1):176–83. doi: 10.1080/13814788.2021.1954155 (PMC8330783; doi:10.1080/13814788.2021.1954155)
Supplement: Supplemental Material: eFigures 2-3 [file IGEN_A_1954155_SM2182.docx]

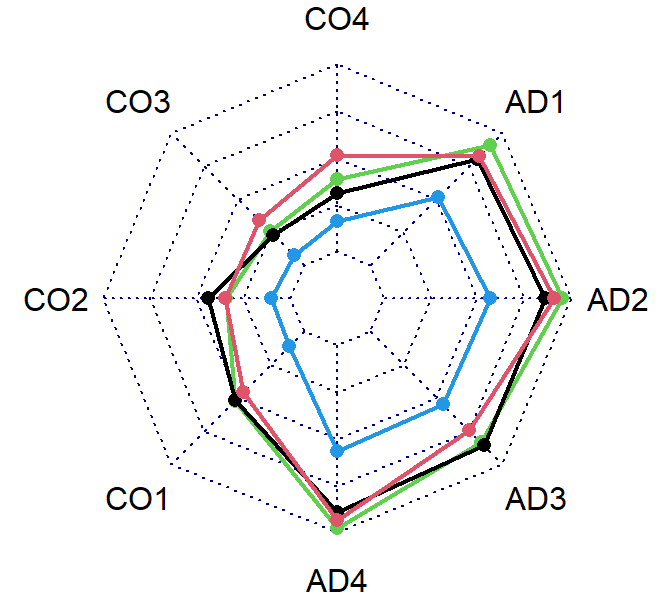


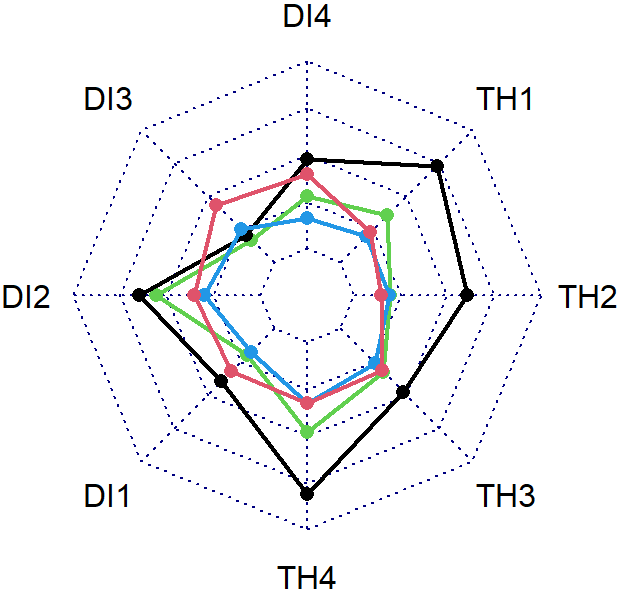


e**Figure 2a**

*Anxiety and depressive symptoms (PHQ-4)*

AD1 Feeling nervous, anxious or on edge

AD2 Not being able to stop or control worrying

AD3 Little interest or pleasure in doing things

AD4 Feeling down, depressed, or hopeless

*Concerns*

CO1 … that I will infect myself

CO2 … that employees will get infected

CO3 … that the practice could be closed (quarantine)

CO4 … about how things will continue economically

**eFigure 2b**

*Threats posed by COVID-19*

TH1 Fear of the SARS-CoV-2 virus is appropriate

TH2 COVID-19 is more dangerous than influenza

TH3 The threat posed by the pandemic is difficult to assess

TH4 I have experienced terrifying courses of COVID-19

*The basic dilemma*

DI1 COVID--19 confronts us with the question of what “price” we are prepared to pay in order to save lives

DI2 The discussion of this question is dangerous

DI3 The discussion of this question is essential

DI4 A satisfactory answer to this question is impossible

eFigures 2a and b
Radar charts showing the **mean values** of the four **subgroup** (**skeptics**, **hardliners**, **balancers**, and **anxious**) regarding anxiety and depressive symptoms and concerns (Figure 1a) and for threat posed by COVID-19 and the basic dilemma (1b). Central values indicate strong agreement, most peripheral values outer strong disagreement


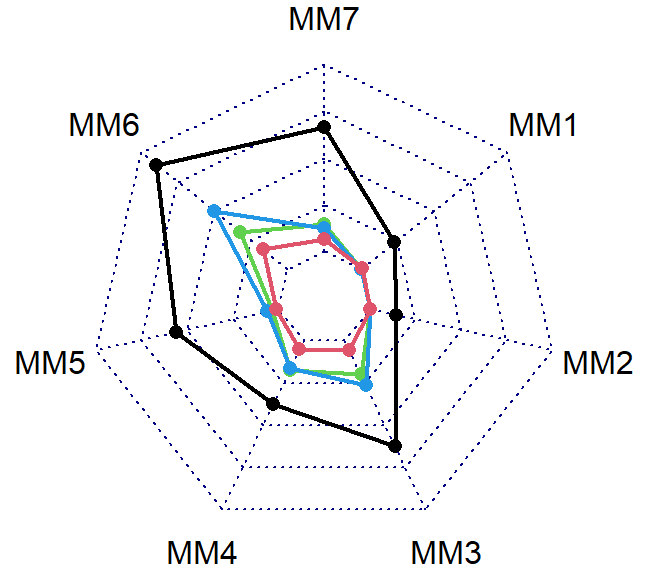


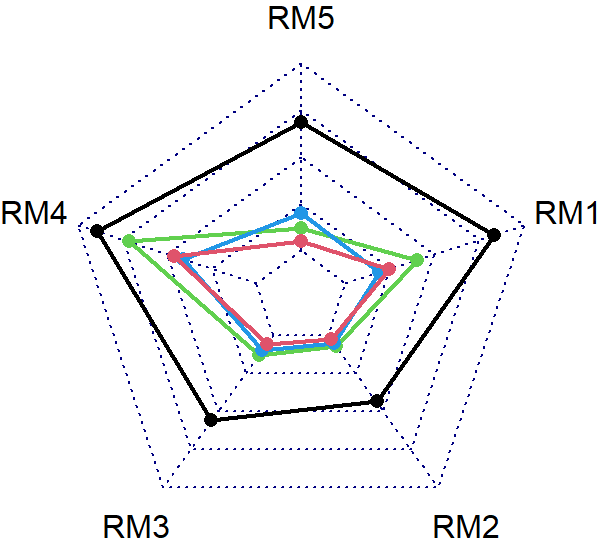


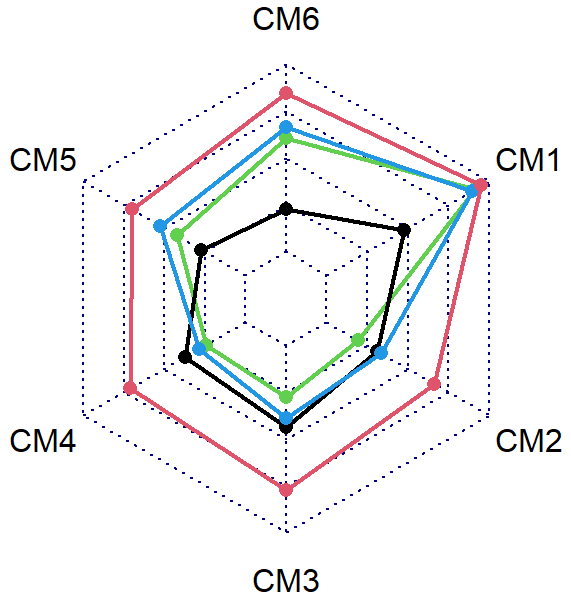


**eFigure 3a**

*Measures taken in March 2020*

MM1 Recommendation to keep your distance

MM2 Ban on major events

MM3 Closure of schools and daycare centers

MM4 Bans on contact in care facilities

MM5 The exit restrictions were understandable in the situation at the time

MM6 From today's perspective, the exit restrictions still seem
 justified

MM7 If no measures had been taken, there could have been many deaths as in northern Italy

**eFigure 3b**

*Relaxation of measures in May 2020*

RM1 I see a significant risk for a "second wave"

RM2 The recommendation to keep your distance is right

RM3 The recommendation to wear face masks is right

RM4 The relaxation of measures happened too quickly

RM5 The pandemic is over and all measures should be stopped das soon as possible (ic)

**eFigure 3c**

*Consequences for the coming months*

CM1 A good preparation for a second wave is necessary not

CM2 In order not to restrict the quality of life of the risk groups too much, pragmatic compromises must be found (which can also cost human lives)

CM3 To protect economy pragmatic compromises ….

CM4 In order to limit the burden on children and young families, pragmatic compromises …

CM5 Coercive measures must be avoided at all costs

CM6 An overreaction to the supposed Covid-19 threat as in the last few months must be avoided in the future

eFigures 3a to 3c
Radar charts showing the **mean values** of the four **subgroup** (**skeptics**, **hardliners**, **balancers**, and **anxious**) regarding measures taken in March 2020 (Figure 2a), relaxations in May 2020 (2b) and consequences for the coming months (2c). Central values indicate strong agreement, most peripheral values outer strong disagreement. For reasons of readability some items were inversely coded or reworded
