# Supplementary material for: The diversity of opinion among general practitioners regarding the threat and measures against COVID-19 – Cross-sectional survey
Source: Eur J Gen Pract. 2021 Jul 28;27(1):176–83. doi: 10.1080/13814788.2021.1954155 (PMC8330783; doi:10.1080/13814788.2021.1954155)
Supplement: Supplemental Material: eFigure 1 [file IGEN_A_1954155_SM2152.docx]

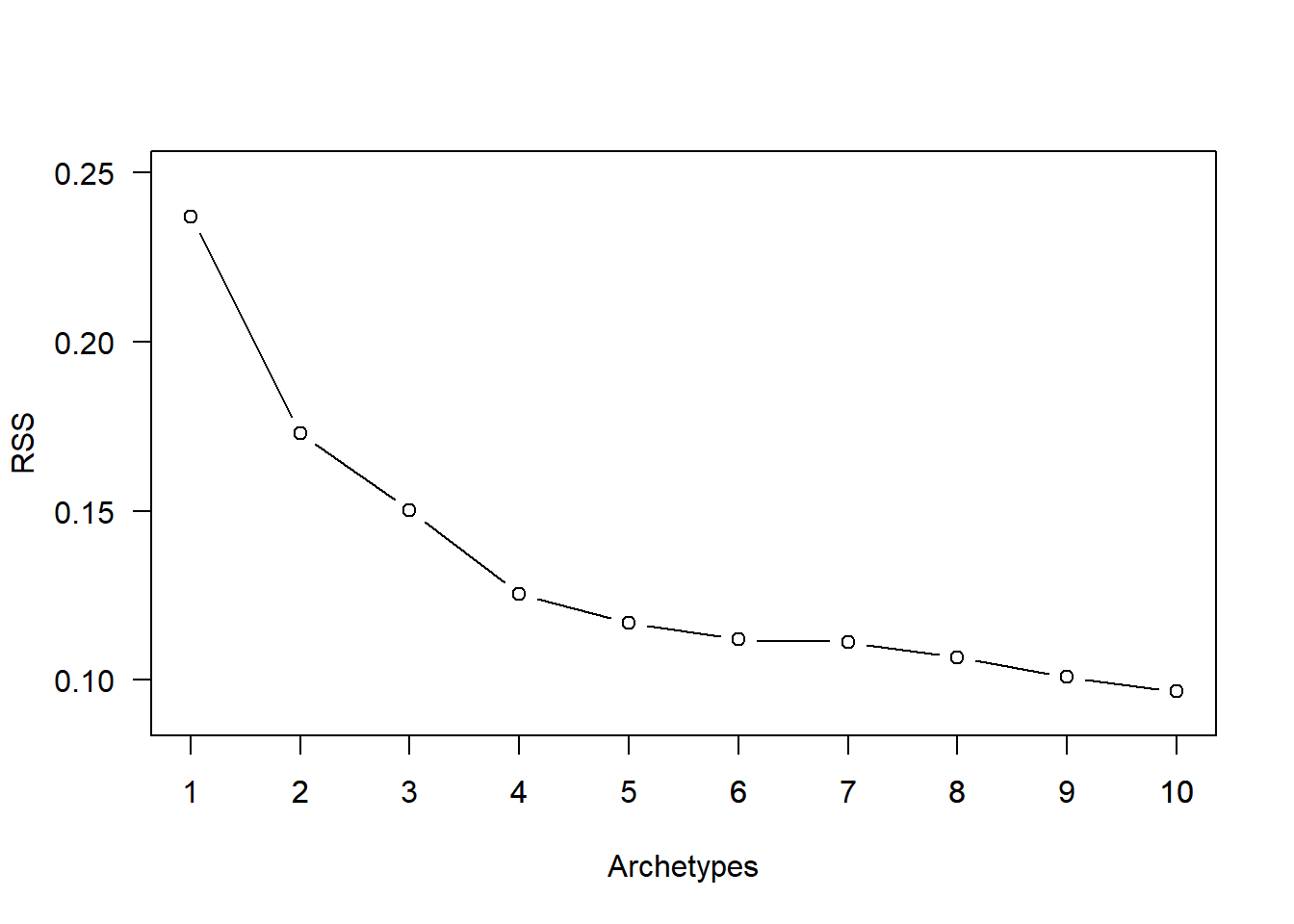


eFigure 1

Residual sum of squares by number of archetypes, indicating that the sum of squared differences between individual observations and the archetypes does not decrease decisively above a number of four archetypes.
